# Beyond conventional approaches: Enhancing photodynamic therapy for refractory feline sporotrichosis caused by *Sporothrix brasiliensis*

**DOI:** 10.1016/j.mmcr.2024.100642

**Published:** 2024-03-06

**Authors:** Mariana Lucy Mesquita Ramos, Vanessa Brito de Souza Rabello, Erica Aparecida dos Santos Ribeiro da Silva, Maria Cristina da Silva Lourenço, Rodrigo Almeida-Paes, Susana Frases

**Affiliations:** a Laboratório de Biofísica de Fungos, Instituto de Biofísica Carlos Chagas Filho, Universidade Federal do Rio de Janeiro, Rio de Janeiro, 21941-902, Brazil; b Laboratório de Micologia, Instituto Nacional de Infectologia Evandro Chagas, Fundação Oswaldo Cruz, Rio de Janeiro, Brazil; c Laboratório de Bacteriologia e Bioensaios, Instituto Nacional de Infectologia Evandro Chagas, Fundação Oswaldo Cruz, Rio de Janeiro, Brazil; d Rede Micologia RJ, FAPERJ, Rio de Janeiro, 21040-360, Brazil

**Keywords:** Sporotrichosis, *Sporothrix brasiliensis*, Feline mycosis, Photodynamic therapy, Antifungal resistance

## Abstract

Male cat, 2 years old, with a refractory infection by Sporothrix brasiliensis, presents a single nodular lesion in the left auricular pavilion. To confirm the diagnosis, cytology, fungal culture, antifungal susceptibility test, molecular analysis, and, to aid in the differential diagnosis, bacterial culture, antibiogram, and histopathology of the lesion were performed. In the absence of therapeutic success with conventional antifungals, photodynamic therapy (PDT) was introduced, demonstrating a satisfactory response in the sixth treatment session.

## 1 Introduction

Sporotrichosis is a subcutaneous mycosis that can present a zoonotic nature highly prevalent in Latin America. It is caused by thermodimorphic fungi belonging to the genus *Sporothrix*. Currently, there are approximately 53 species within the *Sporothrix* genus, with certain species holding clinical significance, as *Sporothrix brasiliensis* [1,2].

However, in last decades, the main mode of transmission has shifted to zoonotic transmission, particularly through scratches or bites from mammalian animals, especially infected domestic cats (*Felis catus*). Over the past two decades, there has been a noticeable increase in the endemic incidence of this mycosis, in almost all regions of Brazil, including Rio de Janeiro, São Paulo, Rio Grande do Sul, Brasília, and Pernambuco, for example [1,[3], [4], [5], [6]].

The clinical manifestation of sporotrichosis in cats varies from cutaneous lesions such as ulcers and nodules to mucosal affections and respiratory conditions. In more severe cases, the fungus can disseminate, in a process depending on the predisposition of the host and *Sporothrix* virulence [1]. The treatment of feline sporotrichosis is limited due to the low availability of antifungal drugs, adverse effects, and high cost. Itraconazole is the drug of choice due to its safety and therapeutic efficiency. Other drugs such as iodides, ketoconazole, amphotericin B, terbinafine, as well as physical methods such as heat-based thermal therapy and surgical removal are also indicated for treating sporotrichosis [7]. However, the emergence of non-wild-type strains to itraconazole complicates clinical responses [8].

The quest for novel alternative treatments becomes imperative in addressing cases of refractory disease and limited available therapeutic choices. Photodynamic therapy (PDT), using the photosensitizer methylene blue, has emerged as a promising avenue for investigation. It has demonstrated compelling efficacy not only in treating tumors but also in combating infections caused by bacteria, parasites, fungi, algae, and viral agents, thereby piquing considerable interest for further research and development [[9], [10], [11]].

*In vitro* studies have demonstrated that the combination of visible light with photosensitizers in the presence of oxygen leads to the generation of reactive oxygen species (ROS), resulting in potent cytotoxic effects. Concurrently, *in vivo* experiments have underscored its clinical effectiveness [[9], [10], [11]].

This study documents a case of *S. brasiliensis* infection in a cat refractory to several conventional treatment approaches. The study highlights the clinical advancement observed in this cat through the implementation of antifungal treatment complemented by low-level laser therapy.

## 2 Case presentation

**Fig. 1:**
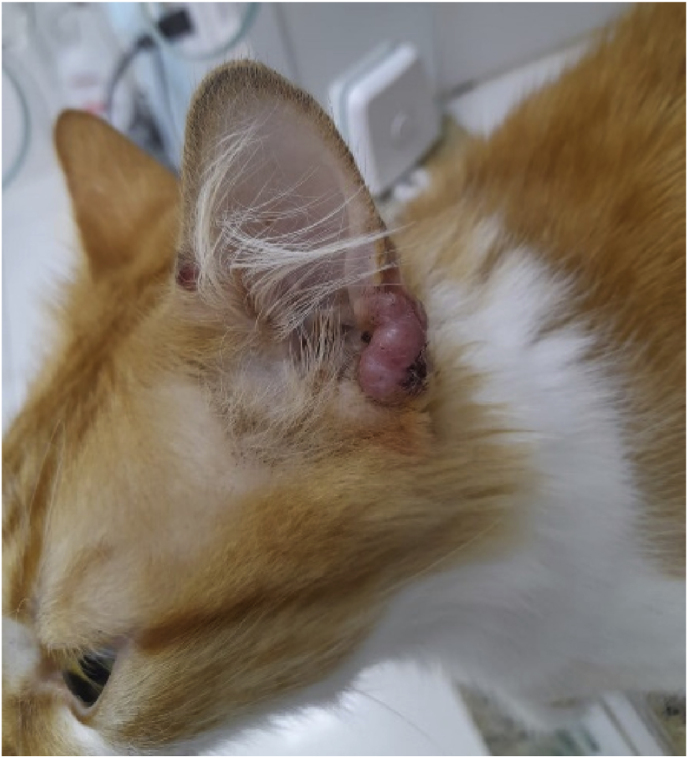
Clinical demonstration of the single nodule at the base of the left ear pinna.

A 2-year-old male cat *(Felis catus),* mixed breed, castrated, and FIV/FELV negative was taken to veterinary care for presenting a single nodular lesion on the left ear pinna. Other areas of the body showed no alterations, as observed in Fig. 1. The cat resided in a high-income neighborhood in RJ, with free-roaming life within a building complex, having contact with other animals and was under the care of animal protectors living in this condominium. The animal was kept hospitalized in the clinic for examinations and treatment. We defined the first day at the clinic as day 0. A biological sample was collected for cytopathology through fine-needle aspiration of the nodule. For fungal culture, material was collected with a swab from the lesion site and placed in a Stuart transport medium container for transportation to the laboratory.

The material was stained with Giemsa eosin Methylene Blue (Merck, Darmstadt, Germany) revealing intracellular (within neutrophils and macrophages) or extracellular round, oval, or cigar-shaped yeast-like structures. These structures exhibited a dark blue color, a pink-stained eccentric nucleus, and a clear halo resulting from membrane retraction. A discreet inflammatory infiltrate composed of intact and degenerated neutrophils (97%), small lymphocytes (2%), and rare vacuolated macrophages was present. In the background, nuclear fibers and red blood cells were identified. Findings are consistent with a fungal infection showing the presence of yeasts associated with a neutrophilic and macrophagic inflammatory process.

**Fig. 2:**
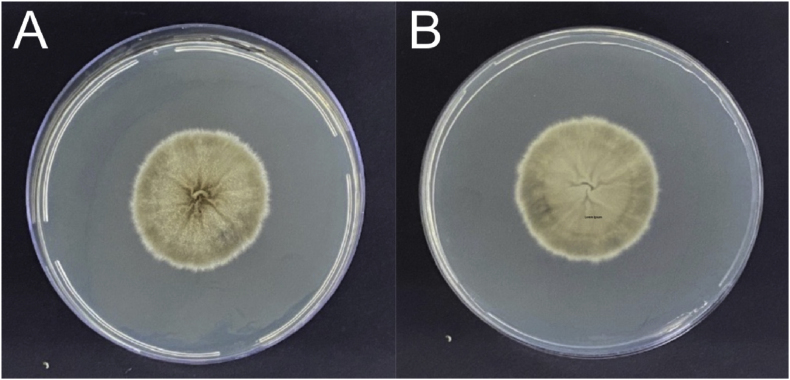
Isolation of fungal colony. Front (A) and back (B) views of the filamentous colonies grown on PDA at 25 °C.

Biological material from the lesions were inoculated on Sabouraud Dextrose Agar with chloramphenicol (Kasvi, Spain) and Mycosel agar (Mycosel Agar, EUA). They were incubated at room temperature (25–30 °C). After ten days of incubation, we observed the growth of membranous colonies, white to cream in color, leathery, with a blackish halo (Fig. 2A), on the hyaline or brownish verse (Fig. 2B). Microscopy analysis revealed hyaline, thin, septate, and branched hyphae with conidiophores of where piriform conidia forming daisy-shaped arrangements. Single cells, predominantly elongated, some ovoid and rounded, are observed. Dimorphism at 37 °C was demonstrated on Brain Heart Infusion Agar (HiMedia, USA). The samples submitted to the reversion process were analyzed microcrocopically and showed pleomorphic yeast structures, ranging from round, oval to cigar-shaped.

Following the diagnosis of *Sporothrix* spp. infection, the animal underwent a therapeutic protocol involving a 30-day administration of itraconazole (100 mg, orally, daily), starting on day two. However, after this conduct period, there was no satisfactory response in the animal's clinical condition. Therapy was changed on day 32 to potassium iodide (10 mg per day, orally) alongside itraconazole. This combination therapy was administered for 30 days, but with limited clinical improvement, leading to the need to increase the potassium iodide dose to 18 mg per day, orally, for an additional 20 days. Due to the presence of secondary infection, amoxicillin with clavulanate (20 mg/kg, orally, every 12 hours, for 20 days) was also used at this point.

The bacterial strain was subjected to MALDI-TOF MS analysis using a Bruker MALDI Biotyper microflex LT (Bruker Daltonics, Bremen, Germany). Sample preparation procedures for MALDI-TOF MS was performed as described by Fraser e colaboradores (2016) [12]. Briefly, a colony was emulsified in 50 μl of 50% ethanol using a sterile, 1 μl inoculating loop, followed by mixing by vortex for 30 s. One microliter of the suspension was then spotted in duplicate onto a 96-spot reusable stainless steel plate which was then allowed until to dry. Dried samples were then overlaid with 1 μl of 70% formic acid and again allowed until to dry. As soon as the sample was dry, they were overlaid with 1 μl of HCCA matrix (Bruker Daltonics). Fully dried plate was subjected to MALDI-ToF MS analysis. Identification was accepted if one of the duplicate spots producing spectra giving a Score ≥2.00. The bacterial strain was identified as *Serratia marcescens* with score 2.18 and 2.00 in each spot.

**Fig. 3:**
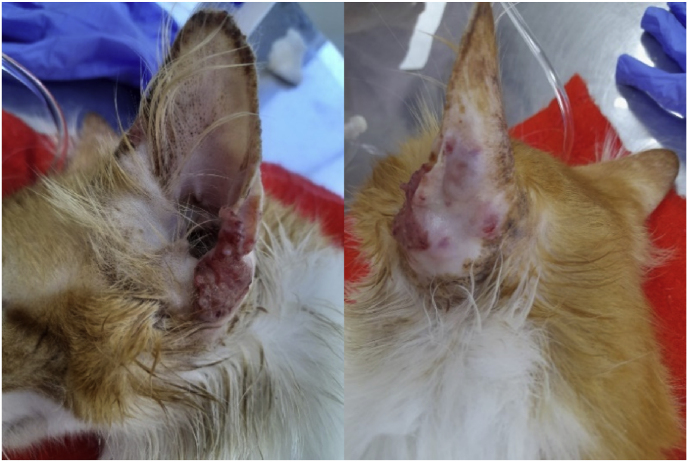
Frontal (A) and dorsal (B) clinical appearance of the left ear pinna after five months of treatment with itraconazole, iodide, and four local injectable applications of amphotericin B.

We also conducted research on *Nocardia* spp. and *Mycobacterium* spp.; nevertheless, the cultures yielded negative results. Despite the treatment attempts and worsening clinical condition, the protocol was modified to include injectable amphotericin B (5 mg/ml and 1% lidocaine) into the lesion of the ear pinna. Four applications were performed with a 21-day interval. At the end of these applications, the clinical condition remained unresponsive, as shown in Fig. 3.

Given the clinical refractoriness to conventional treatments, the animal underwent an anesthetic procedure to collect a fragment from the ulcerated lesion on the ear pinna for a new histopathological examination. A new swab of secretion from the lesion site was also taken for further fungal culture. The swab was sent for fungal culture, molecular identification, and antifungal susceptibility testing of the fungal strain against conventional antifungals.

The fragment sent to the laboratory revealed, in hematoxylin-eosin staining, mild lymphocytic and mastocytic interstitial dermatitis associated with mild chronic folliculitis.

Genomic DNA of the strain was obtained using a previously described protocol [13]. The strain was identified using species-specific primers for main clinical *Sporothrix* species [14]. The species-specific PCR identified strain as *S. brasiliensis*.

Studies to determine their susceptibility to antifungal drugs were conducted to better understand the clinical picture and epidemiological aspects. Antifungal susceptibility test (CLSI, 2008) [15] revealed that the *S. brasiliensis* strain was wild type to all drugs tested, which were ketoconazole, itraconazole and amphotericin B.

**Fig. 4:**
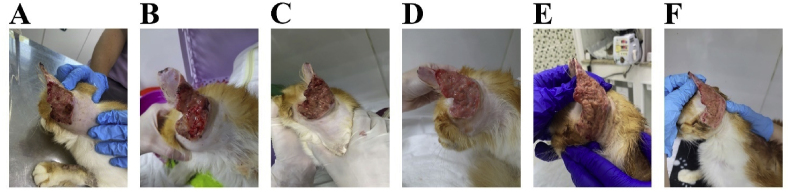
(A**)** Ear damage caused by *S. brasiliensis* on the first day of laser therapy (PDT), (B) second beginning of granulation tissue formation, (C–E**)** third to fifth session, (F) ear healing process.

In the face of extensive auricular injury, we maintained itraconazole therapy along with potassium iodide and Meropenem (20 mg/kg, IV, every 12 hours) for 7 days. With this unsuccessful treatment, we introduced laser therapy as a complementary approach to alleviate the localized condition. The treatment involved Photodynamic Therapy (PDT) with the use of red light associated with a photosensitizer. The laser used was a diode type (InGaAsP, DMC, São Carlos, Brazil), with a power of 100 mW and a wavelength of 660–808nm. The photosensitizer methylene blue (0.01%) was applied to the entire auricular lesion and allowed to act for 10 minutes. The applied radiation dosage was 91,44 J/cm^2^. Six sessions were administered with a 7-day interval between them. As an encouraging sign of progress, the second session revealed the presence of granulation tissue, and by the sixth session, the healing process had started, as illustrated in Fig. 4**.**

Regrettably, after conclusion of the laser therapy, which significantly contributed to clinical improvement in the ear, other new lesions started emerging on the cat's body. Given the unresponsiveness of the animal to systemic therapy and the escalation of the mycosis to different areas of the body, the difficult decision to euthanize the animal became an unavoidable necessity.

## 3 Discussion

Laser therapy demonstrates effects related to immunological processes of photobiological activation and photobiological destruction. In photobiological activation, cells are influenced by low-intensity light, stimulating the immune system. In contrast, photobiological destruction is linked to the destruction of molecular cells through PDT [16]. The study by Yan et al., 2021 also demonstrated the laser's effect on *S. globosa* cells, inducing apoptosis and pyroptosis via NLRP3/Caspase-1, activated by the TH1/TH17 cellular response. This type of treatment aligns with the clinical presentation of the treated feline's ear lesion, as it is believed that laser therapy assisted the immune system in combating the lesion's microorganisms [17].

In 2014, Gilaberte reported a case of sporotrichosis with localized lesions in a human, where PDT was employed in conjunction with the photosensitizer methylene blue [9]. The obtained results were successful. More recently, in 2022, Legabão conducted an overview of *in vitro* and *in vivo* studies on PDT, investigating the photo-inactivation of the complex species of *Sporothrix* [18]. The results showed promising prospects in both humans and animals. During this study, the most extensively tested photosensitizers were methylene blue and aminolevulinic acid. These tests demonstrated fungicidal effects, leading to the conclusion that photodynamic therapy holds significant potential in treating sporotrichosis. This study used methylene blue, with good results [18].

Given the limited availability of antifungal medications and the growing resistance of fungi to these treatments, researchers are exploring the potential photodynamic therapy (PDT) responsiveness of various fungi beyond *Sporothrix* spp. *Candida auris* stands out as an exemplary multidrug-resistant fungus, posing a significant menace to global public health. In a 2021 study by Bapat & Nobile, the efficacy of photodynamic therapy (PDT) was assessed using three distinct light wavelengths: red, green, and blue. These wavelengths were tested both individually and in combination with photosensitizers to combat *C. auris* biofilm formation. The findings yielded promising results, unveiling a potential therapeutic approach for tackling *C. auris* biofilm infections [19].

Laser therapy is commonly employed for the treatment of localized and non-disseminated lesions. Regrettably, following the initial resolution of the ear condition, the animal presented with additional systemic lesions. We speculate that this development might be attributed to prolonged confinement, potentially compromising immune responses due to stress. Furthermore, exposure to environmental sources containing *Sporothrix* sp. could have exacerbated the clinical condition. In instances of such therapeutic challenges, we advocate for the early implementation of physical treatment methods like PDT.

## CRediT author statement

Mariana Lucy Mesquita Ramos: Conceptualization, Methodology, Reviewing and Writing- Original draft preparation.

Iara Bastos de Andrade: Conceptualization, Methodology, Reviewing and Writing- Original draft preparation.

Dario Corrêa-Junior: Conceptualization, Methodology, Reviewing and Writing- Original draft preparation.

Luiza Pereira: Methodology, Investigation, reviewing manuscript.

Rayssa Manrique de Araújo: Methodology, Investigation, reviewing manuscript.

Vanessa Brito de Souza Rabello: Methodology, Investigation, reviewing manuscript.

Andréa Reis Bernardes-Engemann: Methodology, Investigation, reviewing manuscript.

Erica Aparecida dos Santos Ribeiro da Silva: Methodology, Investigation, reviewing manuscript.

Maria Cristina da Silva Lourenço: Methodology, Investigation, reviewing manuscript.

Rodrigo Almeida-Paes: Methodology, Investigation, reviewing manuscript.

Susana Frases: Conceptualization, Methodology, Writing- Reviewing and Editing, Funding acquisition.

## Declaration of competing interest

The authors declare that the research was conducted in the absence of any commercial or financial relationships that could be construed as a potential conflict of interest.
